# Effects of Different Levels of *Lycium ruthenicum* Leaves on Rumen Fermentation, Amino Acids, Fatty Acids and Rumen Bacterial Diversity in Sheep

**DOI:** 10.3390/ani15213118

**Published:** 2025-10-27

**Authors:** Yaya Guo, Jinlong Li, Congbin Xu, Liangzhong Hou, Yuxia Yang, Yan Ma, Yong Tuo, Tongjun Guo

**Affiliations:** 1 Institute of Feed Research, Xinjiang Academy of Animal Science, Urumqi 830011, China; yaya_2025@163.com (Y.G.); lijinlong0120@126.com (J.L.); xcb2318072452@163.com (C.X.); 1890283115@163.com (L.H.); 15022947531@163.com (Y.Y.); myy0129@126.com (Y.M.); t777yvx@126.com (Y.T.); 2 Xinjiang Key Laboratory of Feed Biotechnology, Xinjiang Academy of Animal Science, Urumqi 830011, China

**Keywords:** bacterial diversity, goji berries, lambs, *Lycium ruthenicum* leaves, meat quality, rumen fermentation

## Abstract

**Simple Summary:**

*Lycium ruthenicum* (black goji berry) leaves (LRL) contain a variety of bioactive compounds such as flavonoids, polyphenols, alkaloids, etc., which make it a good candidate for improving the growth performance of livestock and the quality of their products. *Lycium ruthenicum* extracts given to ruminants like sheep have been shown to enhance antioxidant and immune activity and improve ruminal fermentation. However, feeding LRL to ruminants has rarely been reported. In this study, we compared the ruminal microbes, fermentation, and meat quality of F1 generation crosses of Dorper × Hu lambs supplemented with different levels of LRL during the finishing stage. The addition of 5–15% LRL to the sheep diet improved the rumen microbial community, its fermentation activity, and the resulting meat quality.

**Abstract:**

*Lycium ruthenicum* leaves (LRL), as an agricultural by-product rich in bioactive compounds, can be used as an unconventional feedstuff in animal diets and have the potential to improve animal health. This study investigates the effects of dietary supplementation with graded levels of LRL on rumen fermentation, meat amino acid and fatty acid profiles, and rumen bacterial diversity in sheep. Forty three-month-old male Dorper × Hu crossbred F1 lambs with an initial body weight of 29.58 ± 2.06 kg were randomly assigned to four groups (*n* = 10). Over a continuous 63-day trial period, the lambs were fed diets containing 0%, 5%, 10%, and 15% LRL, respectively. At the end of the trial, rumen fluid and longissimus dorsi muscle samples were collected to assess rumen fermentation characteristics, bacterial community structure, and meat quality. The results showed that: (1) The concentrations of acetate, butyrate, and total volatile fatty acids (TVFA) in the rumen were increased in the LRL5% group (*p* < 0.05 or *p* < 0.01). (2) The relative abundance of the phylum Firmicutes and the genus *Ruminococcus* increased (*p* < 0.05), while the relative abundance of the genus *Prevotella* decreased (*p* < 0.05) in the LRL5% group. (3) Meat L* increased (*p* < 0.05), and a* decreased (*p* < 0.05) in the LRL-supplemented groups. (4) The content of sweet amino acids in meat increased in LRL groups (*p* < 0.05). Moreover, the contents of non-essential amino acids, sweet amino acids, and total amino acids in meat increased linearly with increasing dietary LRL levels (*p* < 0.05). (5) Compared with the CON group, the content of C18:0 in meat decreased in the LRL5% group (*p* < 0.05), while the content of C20:1 increased in the LRL10% group (*p* < 0.05). In conclusion, dietary supplementation with LRL can improve meat quality, rumen fermentation, and rumen bacterial community structure in sheep. The recommended dietary inclusion level of LRL ranges from 5% to 15%.

## 1. Introduction

The limited conventional forage resources in China are primarily derived from grasslands, forage crops, agroforestry by-products, and crop straw. Driven by increasing consumer demand for animal products, forage scarcity has emerged as a critical issue for the cattle and sheep industry. Consequently, the development and utilization of unconventional feedstuffs is crucially important for enhancing the economic efficiency of the livestock industry and establishing sustainable farming models.

Botanically classified as *Lycium ruthenicum*, the black goji berry is a perennial, thorny-branched deciduous shrub that falls under the Solanaceae family [1]. It produces fruits rich in bioactive compounds such as anthocyanins and flavonoids, which are active ingredients in many consumer healthcare products [2]. Berry production generates substantial quantities of byproducts including branches, leaves, substandard fruits, fruit stalks, and pomace. In 2023, the cultivation area of *L. ruthenicum* in China reached approximately 10,667 hectares, yielding an estimated 53,000 t of byproducts annually. Among these, LRL accounts for approximately 16,000 t [3]. Nutritional analysis shows that LRL contains 94.90% dry matter, 6.15% crude protein, 11.22% crude ash, 3.14% crude fat, and 1.95% calcium [4]. LRL is also rich in bioactive constituents, including 4.56% total polyphenols, 2.03% total flavonoids, 10.07% polysaccharides, 2.18% free amino acids, and 1.68% betaine [5,6], suggesting its potential for enhancing animal growth performance, antioxidant capacity, and immune function [7,8,9].

Previous research supports the idea that *Lycium* byproducts can be effective nutritional supplements in ruminant feed. Hou et al. [2] found that supplementing the diet of Duolang sheep with 5% substandard *L. ruthenicum* fruits improved feed utilization and rumen fermentation without adversely affecting rumen microbial community composition or richness. Duan et al. [3] reported that adding 3% *Lycium barbarum* (red goji berry) branches and leaves to Hu sheep diets enhanced feed utilization, growth performance, and meat quality with no negative impacts on the rumen. Ju [10] demonstrated that supplementing lamb diets with 0.3% *L. barbarum* polysaccharides improved meat quality while apparently increasing nutrient digestibility and antioxidant function. Additionally, supplementing Tan sheep diets with 4% fermented *Lycium barbarum* pomace improved immune function, antioxidant capacity, slaughter performance, and rumen microbiota composition, thereby promoting growth and development [11].

Compared to *L. barbarum* (red goji berry) byproducts, *L. ruthenicum* (black goji berry) byproducts exhibit higher concentrations of bioactive compounds such as polyphenols, flavonoids, polysaccharides, and vitamin C [12]. Despite this superior bioactive profile, *L. ruthenicum* by-products have not received equivalent research attention. Existing studies indicate that supplementing sheep diets with *L. barbarum* leaves shows potential benefits in product quality and animal health [3]. Therefore, we hypothesize that dietary supplementation with *L. ruthenicum* leaves may likewise exert positive effects on animal health and the quality of livestock products.

An investigation was conducted to assess the impacts of supplementing diets with increasing percentages of LRL on rumen fermentation, muscle amino acid and fatty acid compositional profiles, and bacterial diversity in the rumen of Dorper × Hu crossbred sheep. The findings are expected to provide a theoretical basis for the application of LRL as an unconventional roughage source in sheep production, contributing to more sustainable and environmentally friendly farming practices.

## 2. Materials and Methods

### 2.1. Ethical Statement

The care and use of experimental animals in this study conformed to Chinese guidelines and were carried out under a protocol (Approval code: No. 08 20230508) approved by the Institutional Animal Care and Use Committee of the Xinjiang Academy of Animal Sciences (Urumqi, China).

### 2.2. Materials and Experimental Design

#### 2.2.1. Experimental Materials

**Figure 1:**
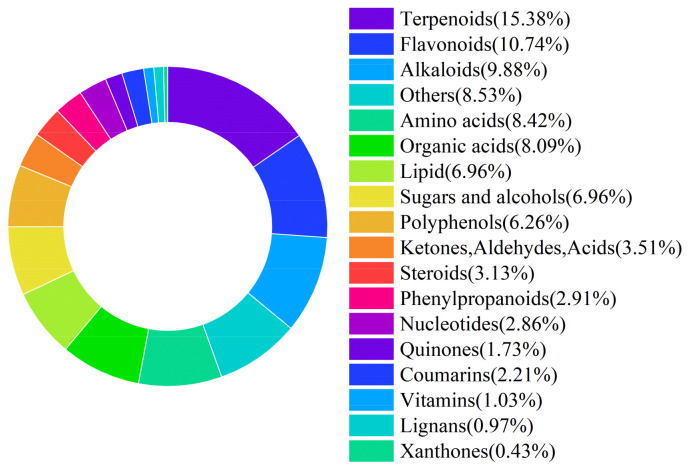
Classification of active substances in LRL.

LRL were provided by Xinjiang Black Goji Berry Biotechnology Co., Ltd. (Korla City, China). The researchers used AOAC method to quantitatively determine the DM, CP, EE, NDF, ADF, Ca and P of LRL [13]. The conventional nutrient composition of LRL is presented in Table 1. Using liquid chromatography-mass spectrometry (LC-MS), a total of 1853 potentially bioactive compounds were detected in LRL. These compounds were classified as follows: 285 terpenoids (15.38%), 199 flavonoids (10.74%), 183 alkaloids (9.88%), 156 amino acids (8.42%), and 150 organic acids (8.09%) (Figure 1).

#### 2.2.2. Experimental Design and Diets

Experiments were conducted at Taihe Ranch, Bachu County, Xinjiang Uygur Autonomous Region, China. A randomized single-factor design was employed. Forty healthy three-month-old male Dorper × Hu crossbred F1 lambs with an average initial body weight of 29.58 ± 2.06 kg were randomly allocated to four groups (*n* = 10 lambs per group). Lambs within each group were group-housed. Four isoenergetic and isonitrogenous experimental diets were formulated based on NRC (2007) [14] nutrient requirements for sheep, differing only in the inclusion level of LRL (0%, 5%, 10%, and 15% of dietary DM). The composition and nutritional levels of the experimental diets are detailed in Table 2. The total trial duration was 63 days, consisting of a 7-day adaptation period followed by a 56-day formal feeding period.

### 2.3. Feeding Management

All experimental animals were managed under identical housing and feeding conditions throughout the trial. Prior to the start of the experiment, lambs were uniformly dewormed and the pens were disinfected. Lambs were fed twice daily at 08:00 and 19:30, with ad libitum access to feed and water. Pens were kept dry and well-ventilated. Daily feed offered was adjusted to ensure approximately 5% orts.

### 2.4. Sample Collection and Measurement of Variables

#### 2.4.1. Slaughter Performance

The body weight of each lamb was recorded immediately prior to slaughter and designated as the slaughter weight (kg). On the day following the end of the feeding trial, after 12 h of feed withdrawal and 2 h of water withdrawal, five lambs per group were randomly selected. The lambs were humanely euthanized according to animal welfare protocols: electrically stunned followed by exsanguination via the carotid artery and jugular vein. After removing the head, feet, skin, and internal organs (with kidneys retained), the hot carcass weight was recorded. Dressing percentage was calculated as (carcass weight/slaughter weight) × 100%. The cross-sectional area of the longissimus dorsi (LD) muscle between the 12th and 13th ribs was exposed. The maximum length (cm) and width (cm) of the LD muscle eye (ribeye) were measured using digital calipers. The ribeye area (cm^2^) was calculated as: [maximum length (cm) × maximum width (cm)] × 0.7. LD muscle samples were collected, stored at −20 °C, and subsequently used for determination of proximate composition, amino acid and fatty acid contents.

#### 2.4.2. Rumen Fluid Sampling and Analysis

After slaughter, approximately 30 mL of rumen fluid was collected from each of the five randomly selected lambs per group directly from the rumen, and the pH was immediately measured using a portable pH meter (Shanghai Yidian Scientific Instrument Co., Ltd., Shanghai, China). Ten mL aliquots of rumen fluid were transferred into cryogenic vials, snap-frozen in liquid nitrogen, and then stored at −20 °C and −80 °C, respectively, for subsequent determination and analysis of rumen fermentation parameters (−20 °C stored) and rumen bacterial diversity (−80 °C stored).

Frozen rumen fluid samples were thawed and centrifuged at 3000× *g* for 10 min at 4 °C. The supernatants were collected for determination of ammonia nitrogen (NH_3_-N) and volatile fatty acids (VFAs) concentrations (acetate, propionate, butyrate, valerate). NH_3_-N concentration was determined using the phenol-hypochlorite colorimetric method [15]. VFAs concentrations were analyzed by high-performance liquid chromatography (HPLC; LC-20AT, Shimadzu, Kyoto, Japan) using 4-methylvaleric acid as an internal standard [16].

Genomic DNA was extracted from rumen fluid samples using the TGuide S96 DNA Kit (Tiangen Biotech Co., Ltd., Beijing, China). The V3-V4 hypervariable region of the bacterial 16S rRNA gene was amplified using universal primers 338F (5′-ACTCCTACGGGAGGCAGCA-3′) and 806R (5′-GGACTACHVGGGTWTCTAAT-3′), with both forward and reverse primers tailed with unique Illumina index sequences for each sample [3]. Polymerase chain reaction (PCR) amplification and mixture preparation were performed according to the method described by Niu et al. [17] in a total volume of 20 µL. Amplified products were purified and quantified. The amplified 16S rRNA gene fragments were sequenced on an Illumina MiSeq platform (Illumina, San Diego, CA, USA) to generate paired-end (2 × 300 bp) reads. Subsequent bioinformatic analysis was conducted following the methods outlined by Li et al. [18].

#### 2.4.3. Meat Quality

LD muscle samples collected between the 12th and 13th ribs were snap-frozen in liquid nitrogen and stored at −20 °C. Meat quality was assessed through routine physical (pH, color, water-holding capacity). Muscle pH was measured at 45 min post-mortem using a portable meat pH meter (Shanghai Yidian Scientific Instrument Co., Ltd., Shanghai, China), calibrated with standard buffers (pH 4.00, 6.86, and 9.18). Meat color parameters [L* (lightness), a* (redness), b* (yellowness)] were measured on the exposed LD surface after 30 min of blooming at 4 °C, using a portable chroma meter (Hangzhou Caipu Technology Co., Ltd., Zhejiang, China) under standardized lighting conditions.

To measure water-holding capacity (drip loss), a 1 cm thick steak was cut perpendicular to the muscle fiber direction. A cylindrical core sample, 3 cm in diameter, was obtained using a coring device (DL-100; Sumspring Sanquan Zhongshi, Jinan, China). The sample was placed between six layers of filter paper (three above and three below) and subjected to a constant pressure of 35 kg for 5 min. The sample weight was recorded before and after pressing. Drip loss was calculated as: [(initial weight − final weight)/initial weight] × 100%.

To determine the proximate LD muscle composition, moisture (method 928.08), crude protein (method 928.08), and crude fat (method 928.08) contents were measured according to standard AOAC methods [13].

#### 2.4.4. Amino Acid Analysis

To determine the amino acid (AA) composition and concentration in frozen LD muscle samples, approximately 100 mg of freeze-dried tissue was homogenized in 1.2 mL of 10% sulfosalicylic acid. The mixture was vortexed thoroughly and centrifuged at 13,500× g for 15 min at 4 °C. The supernatants were collected, filtered through a 0.22 μm membrane filter, and transferred to 2.0 mL glass autosampler vials. Amino acid composition and concentration were determined using a high-speed amino acid analyzer (Model L-8900, Hitachi High-Technologies Co., Tokyo, Japan).

#### 2.4.5. Fatty Acid Analysis and Quantitation

Total fatty acids were extracted from frozen LD muscle samples according to the method described by Junkuszew et al. [19]. Fatty acid methyl esters (FAMEs) were prepared and separated by gas chromatography (GC-450, Varian Co., Walnut Creek, CA, USA). Fatty acid peaks were identified based on retention times compared to known standards (C4-C24 FAME mix; Sigma-Aldrich, Inc., St. Louis, MO, USA). Individual fatty acid concentrations were determined.

### 2.5. Statistical Analysis

Experimental data were initially collated using Microsoft Excel 2016. Statistical analyses were performed using SPSS 26.0 software (IBM Corp., Armonk, NY, USA). Data were subjected to one-way analysis of variance (ANOVA), and differences among treatment means were compared using Duncan’s multiple range test. Orthogonal polynomial contrasts (linear and quadratic) were employed to assess the effects of increasing dietary LRL levels on the measured parameters. Results are presented as the mean ± standard error of the mean (SEM). Significance was defined as *p* < 0.05, and a trend toward significance was defined as 0.05 ≤ *p* ≤ 0.10. For linear and quadratic regression analyses, *p* < 0.05 indicated a significant linear or quadratic response.

For rumen microbiota analysis, representative sequences were clustered into operational taxonomic units (OTUs) at a 97% similarity threshold. The dada2 method in the QIIME2 2020.6 [20] software was used to denoise the sequence. The Silva.138 was used as the reference database to use the naive Bayesian classifier combined with the alignment method to classify the feature sequence. After filtering the low-abundance features (species abundance less than 2), the final feature list was obtained and the number of tags annotated to the species at each level in each sample was counted. The ratio of the number of microbial Reads to the total number of Reads at different levels was used as the relative index for statistics. Alpha diversity indices (ACE, Chao1, Simpson, Shannon) were calculated to estimate species richness and diversity within samples. Beta diversity was assessed using principal coordinates analysis (PCoA) and non-metric multidimensional scaling (NMDS) based on Bray–Curtis dissimilarity matrices to evaluate differences in microbial community structure between groups.

## 3. Results

### 3.1. Effect of LRL on Rumen Fermentation Parameters

As presented in Table 3, the 5% LRL group exhibited higher concentrations of acetate, butyrate, and total volatile fatty acids (TVFA) compared to the control group (*p* < 0.05). Moreover, a highly significant increase in isovalerate concentration was observed in the 5% LRL group (*p* < 0.01). As the dietary inclusion level of LRL increased, the concentrations of butyrate, isovalerate, and TVFA exhibited a quadratic response (*p* < 0.05), initially increasing and then decreasing. No differences were observed among the groups for the remaining parameters (*p* > 0.05).

### 3.2. Effect of LRL on Rumen Bacterial Abundance, Diversity, and Composition

#### 3.2.1. OTU Clustering Analysis Across Groups

**Figure 2:**
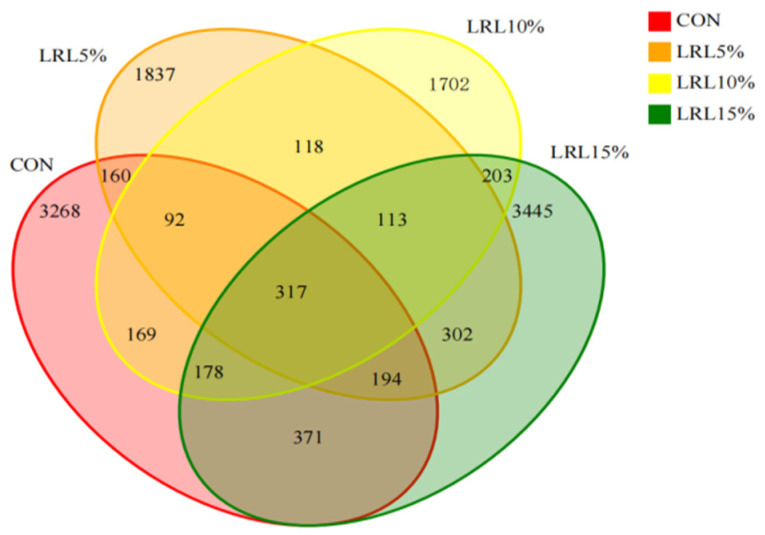
Venn diagram showing the shared and unique rumen bacterial OTUs among all groups. CON, control group; LRL5%, 5% *L. ruthenicum* leaves; LRL10%, 10% *L. ruthenicum* leaves; LRL15%, 15% *L. ruthenicum* leaves. The following figures use the same format and labels.

Supplementary Figure S1 shows the bacterial rarefaction curves. The curves reaching a plateau indicate that the sequencing depth was adequate. Sequencing of the four experimental groups yielded data with coverage exceeding 99%. A total of 1,423,547 paired-end reads were obtained from the rumen fluid samples across all groups. After quality control and merging of paired-end reads, 1,339,075 clean reads were generated. The number of clean reads per sample ranged from 44,935 to 79,085. Clustering sequences at a 97% similarity threshold resulted in a total of 12,469 operational taxonomic units (OTUs) across the four groups. The CON, LRL5%, LRL10%, and LRL15% groups yielded 4749, 3133, 2892, and 5123 OTUs, respectively. A core set of 317 OTUs was shared among all groups. The number of unique OTUs for each group was 3268, 1837, 1702, and 3445, respectively. Compared with the other groups, the LRL15% group possessed the highest number of OTUs (Figure 2).

#### 3.2.2. Analysis of Rumen Bacterial Community Alpha Diversity Indices

As shown in Table 4, the coverage of all groups exceeded 99%, indicating that the sequencing results accurately reflected the rumen microbial community structure. The ACE, Chao1, and Shannon indices in the LRL15% group were significantly higher (*p* < 0.05) than those in the LRL5% and LRL10% groups. No significant differences were observed in the Simpson index among the groups (*p* > 0.05). As the percentage of LRL in the feed increased, the ACE, Chao1, and Shannon indices exhibited a significant quadratic response (*p* < 0.05), initially decreasing and then increasing.

#### 3.2.3. Analysis of Rumen Bacterial Community Beta Diversity

**Figure 3:**
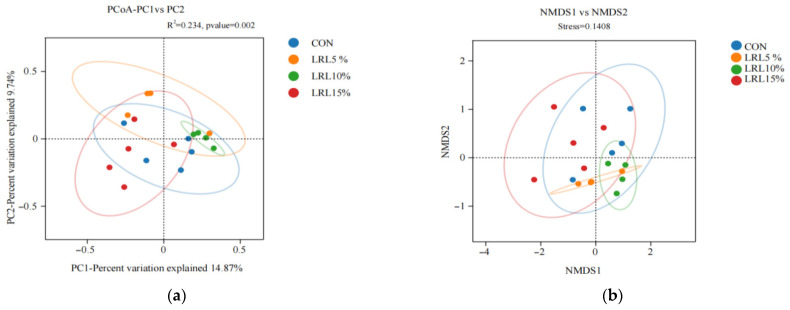
(**a**) Principal coordinate analysis (PCoA) of rumen microbial communities based on Bray–Curtis dissimilarity; (**b**) non-metric multidimensional scaling (NMDS) plot of rumen microbial communities based on Bray–Curtis dissimilarity.

As shown in Figure 3a, the first principal coordinate (PC1) accounted for 14.87% of the total variance, and the second principal coordinate (PC2) accounted for 9.74%. The statistical parameters (R^2^ and *p*-value) for the grouping factor were 0.234 and 0.002, respectively. Samples are colored by group. A larger distance between sample points indicates a greater difference in species composition, whereas a smaller distance indicates greater similarity. The sample points for the LRL10% group were closely clustered, indicating high similarity in microbial composition within this group. Conversely, the sample point spacing of LRL10% and LRL15% groups was greater than the internal spacing of each group. Although there were some differences, they were not significant.

As shown in Figure 3b, non-metric multidimensional scaling (NMDS), a non-linear ordination method, provides a clear visualization of between- and within-group differences, overcoming some limitations of PCoA. An NMDS analysis is generally considered reliable when the stress value is less than 0.2. Closer proximity between samples indicates higher similarity. In this study, the stress value was 0.1408 (<0.2), indicating the reliability of the NMDS ordination. Samples within each group showed high similarity. In particular, there was a separation between the LRL10% and LRL15% groups, but it was not obvious, indicating that the addition of LRL to the diet had a potential effect on the rumen microbial community structure of sheep.

#### 3.2.4. Analysis of Rumen Bacterial Composition and Community Structure

**Figure 4:**
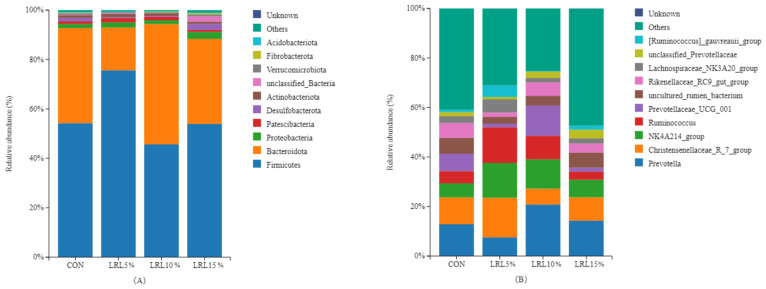
Distribution of bacterial taxa averaged at the phylum level (**A**) and genus level (**B**) across the different treatment groups (values represent percentage of total sequences).

Firmicutes and Bacteroidota are the dominant bacterial phyla across all groups (Figure 4A). The relative abundance of Firmicutes in the rumen of the LRL5% group was significantly higher than that in the LRL10% group (*p* < 0.05), while the relative abundance of Bacteroidota was significantly lower than that in the other experimental groups (*p* < 0.01) (Table 5). The relative abundance of Desulfobacterota in the LRL15% group was significantly higher than that in the LRL5% and LRL10% groups (*p* < 0.05). As the percentage of dietary LRL increased, the relative abundances of Firmicutes and Desulfobacterota exhibited a significant quadratic response (*p* < 0.05), initially increasing and then decreasing. No significant differences were observed in the relative abundances of the other bacterial phyla (*p* > 0.05).

As shown in Figure 4B and Table 6, at the genus level, the relative abundance of *Christensenellaceae_R-7* in the LRL5% group was significantly higher than that in the LRL10% group (*p* < 0.01) and the LRL15% group (*p* < 0.05). The relative abundance of *NK4A214* in the LRL5% group was significantly higher than that in the control and LRL10% groups (*p* < 0.01), and significantly higher than that in the LRL15% group (*p* < 0.05). The relative abundance of *Ruminococcus* in the LRL5% group was significantly higher than that in the control and LRL15% groups (*p* < 0.05). The relative abundance of *Lachnospiraceae_NK3A20* in the LRL5% group was significantly higher than that in the control and LRL10% groups (*p* < 0.05). In contrast, the relative abundance of *Prevotella* was significantly lower than that in the LRL10% group (*p* < 0.05), and the relative abundance of *Rikenellaceae_RC9_gut* was significantly lower than that in the control and LRL15% groups (*p* < 0.05). As the dietary LRL inclusion level increased, the relative abundances of *NK4A214*, *Ruminococcus* and *Lachnospiraceae_NK3A20* exhibited a quadratic response (*p* < 0.05), initially increasing and then decreasing. No differences were observed in the relative abundance of the other bacterial genera (*p* > 0.05).

**Figure 5:**
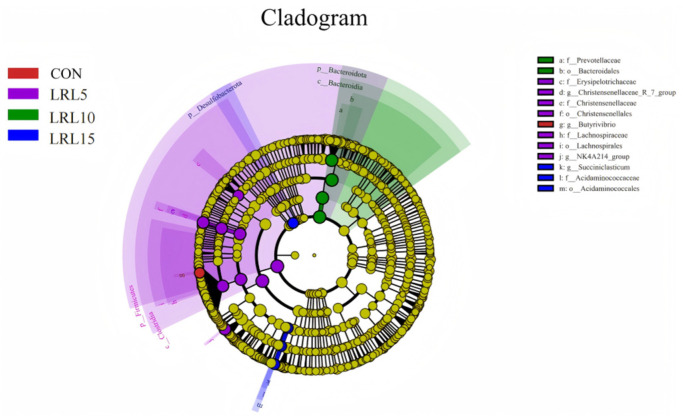
LEfSe cladogram comparing microbial communities among the 4.0 elevations.

Linear discriminant analysis effect size (LEfSe) was employed to identify differentially abundant bacterial taxa across the groups. The representative cladogram illustrates the most significant differences in microbial community structure from phylum to species level among the four experimental groups (Figure 5). The results showed that one taxon was enriched in the CON group, nine taxa were enriched in the LRL5% group, three taxa were enriched in the LRL10% group, and four taxa were enriched in the LRL15% group.

**Figure 6:**
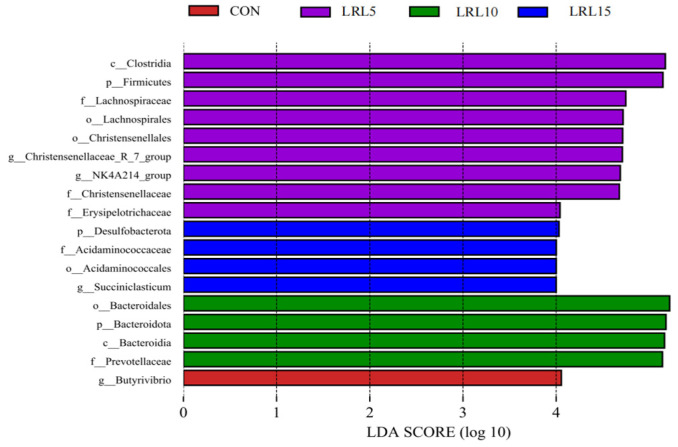
Histogram of LDA scores calculated for each taxon from phylum to genus.

The histogram of LDA scores (Figure 6) displays the specific taxa with significant abundance differences between the CON, LRL5%, LRL10%, and LRL15% groups. The results indicate that the taxa Firmicutes, *Clostridia*, Lachnospirales, Lachnospiraceae, Christensenellaceae, Christensenellales, *Christensenellaceae_R-7_*group, *rumen_bacterium_NK4A214*, *NK4A214_*group, *Ruminococcus_bromii*, and Erysipelotrichaceae exhibited the most significant enrichment in the LRL5% group. In the LRL10% group, *Succiniclasticum*, *Succiniclasticum_ruminis*, Acidaminococcales, Desulfobacterota and Acidaminococcaceae were more abundant. The LRL15% group was characterized by greater abundance of Bacteroidota, Bacteroidia, Bacteroidales, Prevotellaceae and *Prevotella_ruminicola*.

### 3.3. Effect of LRL on Slaughter Performance and Meat Quality of Lambs

#### 3.3.1. Slaughter Performance and Proximate Meat Composition

As shown in Table 7, meat lightness (L) was significantly increased in the LRL5% group (*p* < 0.05) and significantly increased in the LRL10% and LRL15% groups (*p* < 0.01) compared with the control group. Meat redness (a) was highly significantly decreased (*p* < 0.01) in all LRL-supplemented groups. No differences were observed among the groups for the remaining parameters (*p* > 0.05).

#### 3.3.2. Effect of LRL on Amino Acid Content in the Longissimus Dorsi Muscle of Lambs

As shown in Table 8, compared to the control group, the Pro concentration was highly significantly increased (*p* < 0.01) in the LRL-supplemented groups, while Thr, Arg, and SAA were significantly increased (*p* < 0.05). Thr was highly significantly increased in the LRL15% group (*p* < 0.01), and the DAA content in the LRL5% and LRL10% groups was significantly higher than that in the LRL15% group (*p* < 0.05). Gly in the LRL10% and LRL15% groups was highly significantly higher than that in the control group (*p* < 0.01) and significantly higher than that in the LRL5% group (*p* < 0.05). The NEAA content in the LRL10% group was significantly higher than that in the control group (*p* < 0.05), and His concentration was significantly higher than that in the LRL15% group (*p* < 0.05). Ala content in the LRL15% group was significantly higher than that in the LRL10% group (*p* < 0.05) and highly significantly higher than that in the control group (*p* < 0.01). Lys in the LRL15% group was significantly higher than in the control group (*p* < 0.05), while Glu was highly significantly lower than in the other groups (*p* < 0.01).

As the dietary percentage of LRL increased, the concentration of Gly, Ala, Ser, Pro, Val, Thr, Lys, Arg, NEAAs, SAAs, and TAAs showed a linear increase (*p* < 0.05). Asp exhibited a quadratic response (*p* < 0.05), initially decreasing and then increasing, while Glu and DAA showed a quadratic response (*p* < 0.05), initially increasing and then decreasing. No differences were observed for the remaining parameters (*p* > 0.05).

#### 3.3.3. Effect of Black Goji Berry Leaves on Fatty Acid Content in the Longissimus Dorsi Muscle of Lambs

As shown in Table 9, compared with the control group, the concentration of C18:0 in the meat of the LRL5% group was reduced, while the content of C20:1 in the meat of the LRL10% group was significantly higher than that of the control group (*p* < 0.05). There were no significant differences in the other indicators among the groups (*p* > 0.05).

## 4. Discussion

### 4.1. Effect of Lycium ruthenicum Leaves on Rumen Fermentation Parameters

The acid–base balance within the rumen is a critical factor for maintaining the overall health of ruminants. Excessively low ruminal pH can lead to ruminal acidosis, which not only inhibits the activity of rumen microbes, but also adversely affects the animal’s appetite and nutrient absorption capacity [21]. The concentration of ammonia nitrogen in the rumen reflects the efficiency of dietary protein and carbohydrate breakdown and utilization by the host animal [22]. The normal pH range in sheep rumen fluid is 5.5–7.0, and the normal range for NH_3_-N concentration is 5.0–30.0 mg/dL [3]. In the present study, the ruminal pH and NH_3_-N concentration in all LRL-supplemented groups remained within these normal ranges. This indicates that dietary supplementation with LRL had no adverse effects on rumen fermentation, which is consistent with previously published studies. The gradual increase in NH_3_-N concentration with increasing dietary LRL levels might be attributable to the bioactive flavonoids and alkaloids present in LRL. These compounds could modulate the ruminal environment and nitrogen utilization by the host, suggesting that the observed increase in NH_3_-N concentration may be associated with altered nitrogen metabolic efficiency [10].

Volatile fatty acids (VFAs), primarily acetate, butyrate, isovalerate, and valerate, are short-chain fatty acids produced by rumen microbial metabolism of cellulose and hemicellulose. Their production rates influence nutrient metabolism and play a vital role in the animal’s nutrition and energy supply [22,23]. Acetate and butyrate can be interconverted within the rumen, thereby influencing the energy metabolism of ruminants. The acetate-to-propionate ratio is often used as an indicator of energy utilization efficiency, with a lower ratio suggesting higher efficiency. Increases in branched-chain VFAs like isovalerate can influence butyrate concentration [24]. Su et al. [25] reported that supplementing diets of meat sheep with *Allium mongolicum* Regel powder, which contains bioactive substances such as phenols, flavonoids, and polysaccharides, increased the concentrations of acetate and TVFA in the rumen, improved rumen fermentation, and enhanced nutrient utilization, consequently improving growth performance. In our study, the 5% LRL supplementation level increased the concentrations of acetate, butyrate, and TVFA, and highly increased isovalerate concentration, which aligns with the findings of Duan [26]. Furthermore, we observed a quadratic response in the concentrations of acetate, butyrate, and TVFA to increasing dietary LRL levels, with the peak occurring at the 5% inclusion level, followed by a decline at higher levels (10% and 15%). This pattern may be attributed to the bioactive plant compounds present in LRL. At appropriate concentrations, polyphenolic compounds can enhance the activity of fibrolytic bacteria in the rumen, whereas excessive concentrations may inhibit the growth of ruminal microorganisms [27]. This is consistent with the changing trends observed for fibrolytic bacteria such as the *Christensenellaceae_R_7_*group, *NK4A214_*group, *Lachnospiraceae_NK3A20_*group and *Ruminococcus* in Table 6. Such selective modulation likely creates a more efficient environment for the degradation of fibrous material, thereby leading to increased production of acetate, butyrate, and TVFA. Additionally, as the dietary inclusion of LRL increased, the NDF and ADF content of the diet also rose. At higher inclusion levels, this dietary fiber displaced more readily fermentable concentrates, which may have contributed to an overall reduction in the fermentation rate. Consequently, the quadratic response in acetate, butyrate, and TVFA concentrations with increasing LRL levels can be explained by the combined effects of these factors. No significant differences were observed in the acetate-to-propionate ratio among groups, and all ratios were greater than 3:1, indicating that an acetate-type rumen fermentation pattern predominated. This effect could be attributed to active components in LRL, such as flavonoids, polysaccharides, and alkaloids. These compounds may enhance TVFA concentration and branched-chain amino acid metabolism by providing specific metabolic precursors, modulating the populations of fibrolytic and proteolytic bacteria, and optimizing the microbial utilization efficiency of precursors for acetate and butyrate [3]. This promotes the degradation of fibrous material and protein components in the rumen, improving the host’s utilization of nutrients and enhancing growth performance [28]. In summary, dietary supplementation with *Lycium ruthenicum* leaves can modulate the ruminal fermentation environment.

### 4.2. Effect of Lycium ruthenicum Leaves on Rumen Microbial Diversity in Lambs

The rumen is a vital organ for digestion, absorption, and metabolism in ruminants. Rumen microbes, serving as a crucial source of protein and energy for the host, have a direct impact on production performance and meat quality through their community structure and function [29]. Microbial diversity is regulated by factors such as breed, age, diet, and environment. In alpha diversity analysis, the ACE and Chao1 indices reflect species richness, while the Shannon and Simpson indices reflect diversity and evenness; these are key metrics for assessing rumen microbial diversity [30,31]. Liu et al. [32] found that adding niacinamide to the diet of lactating dairy cows increased the Chao1 index. Xiao et al. [33] reported that supplementing the diet of Yunling cattle with mulberry leaf powder elevated the ACE and Chao1 indices, thereby optimizing the relative abundance of cellulolytic bacteria in the rumen and enhancing the degradation of dietary fiber. In this study, the coverage of all samples exceeded 99%, indicating that the sequencing results accurately reflected the microbial diversity within the sheep rumen. While no significant differences in alpha diversity indices were observed between the control group and the LRL-supplemented groups, the ACE, Chao1, and Shannon indices were lower in the LRL5% and LRL10% groups compared with the LRL15% group. The Simpson index did not differ among groups. These results support our hypothesis that dietary supplementation with LRL can modulate the richness and diversity of the ruminal bacterial community. The flavonoids and polyphenolic compounds present in LRL may inhibit the over-proliferation of microbes by suppressing pathogenic microorganisms and modulating inter-microbial interactions [26]. The specific mechanisms involved require further investigation.

The phyla Bacteroidota and Firmicutes play dominant roles in the rumen environment. Firmicutes constitute a major phylum involved in the degradation of fibrous material in the rumen. The phylum primarily consists of fibrolytic genera such as *Ruminococcus* and *Butyrivibrio*, as well as lactate-producing genera like *Lactobacillus*. Bacteroidota is another key phylum that promotes the breakdown of proteins and plant polysaccharides and also participates in the degradation of some fibrous materials [34]. The genus *Prevotella* is primarily involved in degrading nutrients such as starch, cellulose, and protein, directly participates in carbohydrate digestion, and can alter rumen fermentation patterns. An increase in its relative abundance is beneficial for the decomposition and utilization of fibrous plant material [35,36]. The genus *Ruminococcus* specifically degrades structural polysaccharides like lignocellulose and arabinoxylan by secreting cellulase and xylanase complexes. Its key metabolic end products include precursors for short-chain fatty acids (SCFAs) such as succinate and acetate, accompanied by the generation of intermediate products like formate and lactate. An increase in its relative abundance may be associated with enhanced crude fiber degradation rate and SCFA production, thereby influencing the ruminal fermentation environment and the host’s nutrient utilization efficiency [37]. Guo et al. [38] demonstrated that adding 3% soybean oligosaccharides, chitosan oligosaccharides, or mannan oligosaccharides to the diet improved the rumen fermentation environment, increased the relative abundances of Firmicutes, Bacteroidota, and *Ruminococcus* in Holstein calves, and enhanced the utilization efficiency of dietary energy and fiber nutrients. Mu [39] found that grape seed proanthocyanidins could modulate the rumen fermentation environment and the relative abundance of *Prevotella* in the intestinal microbiota of sheep, thereby improving growth performance.

In our study, the LRL5% group exhibited a significant decrease in Bacteroidota and a significant increase in Firmicutes. This indicates that dietary LRL supplementation can increase the relative abundance of Firmicutes in the sheep rumen, potentially enhancing the secretion of digestive enzymes by the host. It may also promote the generation of SCFAs like acetate and butyrate in the rumen by modulating the relative abundance of Bacteroidota, thereby improving the degradation rate of fibrous material and regulating the host’s energy metabolism and the utilization efficiency of nutrients like cellulose [40,41]. The relative abundance of *Ruminococcus* increased, while that of *Prevotella* decreased in the LRL5% group, suggesting that dietary LRL supplementation can enhance energy metabolic rate by promoting VFAs production. This effect might be related to the potential impact of LRL on rumen fermentation and nutrient metabolism. The increased relative abundance of *Ruminococcus* could be associated with enhanced nutrient utilization and VFAs production, influencing the overall rumen fermentation pattern and nutrient efficiency [37].

In addition, β diversity analysis showed that there were some differences in rumen microbial community structure between LRL10% and LRL15% groups, which may be due to the different addition levels of LRL in the diet, that is, the higher the content of LRL, the higher the bioactive substances such as polyphenols or flavonoids in the diet, so as to better improve the rumen bacterial community of the experimental sheep [2]. This was consistent with the results of α diversity, that is, compared with the LRL10% group, the bacterial richness (ACE and Chao1 index) and diversity (Shannon index) of the LRL15% group were higher than those of the LRL10% group. In the LRL15% group, Desulfobacterota was enriched at the phylum level and *Prevotella_ruminicola* was identified at the species level by LEfSe analysis. This phenomenon may represent that the rumen bacterial community of sheep is adapted to the bioactive substances in the LRL.

In this study, dietary LRL supplementation modulated the concentration of VFAs in the sheep rumen, consequently improving the relative abundance of microbes associated with cellulose degradation. The bioactive compounds in LRL, such as polysaccharides, flavonoids, and alkaloids, may increase the relative abundance of specific bacterial taxa and alter the microbial community structure, thereby affecting the growth and activity of particular microbial groups in the rumen [42]. In conclusion, dietary supplementation with *Lycium ruthenicum* leaves can modulate the diversity and composition of dominant bacteria in the ruminal bacterial community of sheep. The specific mechanisms underlying these effects warrant further research.

### 4.3. Effects of Lycium ruthenicum Leaves on Slaughter Performance and Meat Quality in Sheep

#### 4.3.1. Effect of *Lycium ruthenicum* Leaves on Slaughter Performance and Proximate Meat Composition

For meat-producing animals, slaughter performance is primarily evaluated through two indicators, carcass weight and dressing percentage, which reflect the efficiency of meat production. Eye muscle area measures the cross-sectional area of the longissimus dorsi muscle in pigs or sheep post-slaughter and is often positively correlated with the animal’s meat yield [43]. Ju [10] reported that adding 0.3% *Lycium barbarum* polysaccharides to lamb diets had no significant effect on carcass weight, dressing percentage, or eye muscle area, which is consistent with the results of the present study. The nonsignificant difference in carcass weight also corroborates the nonsignificant difference in eye muscle area, indicating that dietary supplementation with an appropriate level of LRL has no negative impact on the slaughter performance of sheep. The dressing percentage value in the 5% LRL group was higher than in the other experimental groups, and it also increased the eye muscle area of the sheep. This could be because supplementing sheep diets with an appropriate amount of LRL modulates the ruminal fermentation environment and the relative abundance of cellulolytic bacteria, thereby promoting the absorption and utilization of dietary nutrients and consequently improving sheep growth and meat production performance.

Indicators such as pH and meat color reflect the quality of animal meat. Meat pH is influenced by the breakdown of glycogen in muscle, which produces lactic acid. Meat color reflects the freshness of muscle quality, which can be judged intuitively by the naked eye [44,45] and influences consumer purchasing decisions. The a* value is directly related to the content of myoglobin and hemoglobin in the muscle, while a higher L* value indicates a desirable lighter meat color. The color of the longissimus dorsi muscle can serve as a reference indicator for carcass meat color, but it is important to note variations in color between different muscle groups. It is influenced by measurement methods, muscle quality, fat content, and other factors, and therefore cannot completely replace the assessment of overall carcass meat color [46].

Zhao et al. [47] found that adding 0.4% *Lycium barbarum* polysaccharides to the diet of Rex rabbits had no significant effect on muscle pH, which aligns with our findings. Likewise, Su et al. [48] reported that adding 400 mg/kg of mulberry leaf flavonoids to the diet of meat sheep had no significant effect on meat color. Li et al. [49] observed that adding 0.2% sea buckthorn flavonoids to the diet of broilers reduced the fat content and increased the protein content in the meat. In this study, the L* value was highly significantly increased, and the a* value was highly significantly decreased in the LRL-supplemented groups. This could be attributed to polyphenolic bioactive substances in LRL, such as anthocyanins and flavonoids, which may enhance meat lightness, make the meat appear brighter, reduce the myoglobin content in the meat, and modulate the body’s metabolism of pigments [50].

#### 4.3.2. Effect of *Lycium ruthenicum* Leaves on Amino Acid Content in the Longissimus Dorsi Muscle of Lambs

Mutton is a livestock product known for its low fat, high protein content, and distinctive gamey flavor. The nutritional value of its protein is directly related to the types, quantities, and proportions of its fundamental building blocks—amino acids. The content and composition of essential amino acids in meat are crucial factors influencing the nutritional value of mutton. Certain amino acids contribute to flavor formation through self-modification or synergistic interactions with lipids, undergoing processes like sulfhydryl oxidation, aromatic hydroxylation, and carbonyl formation to produce flavorful compounds like aldehydes and ketones. Specifically, aspartic acid, glutamic acid, glycine, alanine, proline, and arginine are precursor substances for meat flavor formation, directly influencing the *umami* taste of muscle and are thus termed *umami* amino acids [51,52]. Lysine enhances the activity of antioxidant enzymes, improving the body’s antioxidant capacity, promoting growth and development, and maintaining health; it can also improve mutton flavor after heat treatment. Arginine can enhance immunity by stimulating the proliferation of white blood cells and macrophages and helps maintain the balance between energy intake and expenditure. Serine primarily participates in lipid metabolism and immune system function [53,54]. Shao et al. [55] found that adding tender sea buckthorn leaves and branches, which contain flavonoids, to broiler diets increased the amino acid content in chicken meat, thereby improving its nutritional value. Yun et al. [56] reported that adding grape seed anthocyanins to the diet of Hainan black goats increased the content of essential amino acids and total amino acids in the longissimus dorsi muscle, with sweet amino acids like lysine, threonine, and serine showing varying degrees of increase, thus improving meat flavor and nutritional value.

Li et al. [57] demonstrated that adding 10% pepper straw, containing bioactive substances like flavonoids, to the diet increased the contents of total amino acids, essential amino acids, umami amino acids, and sweet amino acids in mutton. In this study, dietary supplementation with LRL increased the contents of umami amino acids, sweet amino acids, and non-essential amino acids. Furthermore, essential amino acids and total amino acids showed a linear increasing trend, with the LRL5% group exhibiting the highest levels of umami amino acids, sweet amino acids, and essential amino acids. This could be because plant-wide targeted metabolomics in this study detected 156 types of amino acids in LRL, supplementing the diet with abundant alkaloids and pharmacologically active amino acids, allowing more amino acids to enter the bloodstream and promoting the deposition of umami amino acids in the meat. Another possibility is that bioactive substances in LRL, such as anthocyanins, flavonoid polyphenols, and alkaloids, modulate the concentration of butyrate in the sheep rumen, improve energy metabolism, promote the deposition of amino acids in the meat, and further enhance the nutritional value and flavor of mutton.

#### 4.3.3. Effects of *Lycium ruthenicum* Leaves on Fatty Acid Content in the Longissimus Dorsi Muscle of Lambs

As an important source of dietary fat for humans, the fatty acid composition of livestock and poultry meat primarily includes saturated fatty acids (SFAs), monounsaturated fatty acids (MUFAs), and polyunsaturated fatty acids (PUFAs). Typically, the fatty acid content in animal muscle follows the order: SFAs > MUFAs > PUFAs. MUFAs can lower blood sugar and cholesterol levels; PUFAs can reduce cholesterol levels and ameliorate conditions like inflammation and cardiovascular diseases; excessive intake of SFAs increases the risk of cardiovascular diseases [58,59]. Alpha-Linolenic acid, linoleic acid, and arachidonic acid are essential fatty acids for animals, and their deficiency can cause various diseases [60]. Stearic acid is a major component contributing to the mutton odor; the higher its concentration, the stronger the mutton odor. C20:1 is a MUFA that not only reduces serum cholesterol and low-density lipoprotein content and regulates energy metabolism, but also participates in modulating cell signaling and inflammatory responses [61]. Li et al. [57] found that adding pepper straw containing alkaloids to the diet of Dorper × Hu crossbred lambs had no significant effect on palmitic acid, unsaturated fatty acids, or polyunsaturated fatty acids in the meat. Jiao [62] reported that adding 5% *Lycium barbarum* (red goji berry) pomace to sheep diets reduced the content of stearic acid among the UFAs, reduced the strong mutton flavor, and enhanced its nutritional value. In our study, the stearic acid content was reduced in the LRL5% group, indicating that LRL supplementation can decrease the mutton odor. This could be because bioactive substances like flavonoids and alkaloids in LRL enhance the body’s antioxidant properties, reduce the oxidative deterioration of unsaturated fatty acids, thereby improving the nutritional value of mutton, reducing the mutton odor, and making it more aligned with modern consumer dietary preferences [63,64]. Another possibility is that bioactive compounds like polyphenols and flavonoids in LRL can modulate the acetate concentration in the rumen, which can be catalyzed by fatty acid synthase to synthesize long-chain fatty acids. Simultaneously, enzymes like lipoprotein lipase and hormone-sensitive lipase can hydrolyze protein-bound triglycerides and triglycerides in adipose tissue into glycerol and free fatty acids, thus regulating the fatty acid composition [65]. Dietary supplementation with LRL also promoted the synthesis of C20:1. This might be related to LRL’s bioactive substances, such as polyphenols and flavonoids, modulating the activity of lipogenic enzymes. Cis-11-Eicosenoic acid can be oxidized to provide energy when energy demand increases, maintaining normal bodily functions. Furthermore, previous research has demonstrated that plant polyphenols can modulate ruminal fatty acid metabolism, primarily through partial inhibition of the ruminal biohydrogenation process by polyphenolic compounds, thereby promoting the accumulation of MUFAs and conjugated linoleic acid (CLA) [63,66]. This provides a plausible explanation consistent with our findings, where the observed increase in C20:1 content and decrease in C18:0 may reflect a partial inhibition of the biohydrogenation pathway. The decreased content of PUFAs in the LRL group may be related to changes in the rumen microbial community. The bioactive compounds in LRL may selectively inhibit certain biohydrogenating bacteria, thereby altering the content of polyunsaturated fatty acids entering the post-ruminal digestive tract and thus affecting the animal’s post-absorptive lipid metabolism. The underlying mechanism may involve interactions between flavonoids or polyphenolic compounds in LRL and rumen microbes, potentially influencing the biohydrogenation process. However, this hypothesis requires further validation through future studies specifically designed to investigate ruminal lipid metabolism and microbial population dynamics.

## 5. Conclusions

The results of this trial indicate that dietary supplementation with LRL can improve the rumen fermentation environment in Dorper × Hu crossbred lambs, while having no negative impact on the structure and richness of the rumen microbial community and increasing the content of amino acids and fatty acids in the meat, thereby improving meat quality. Under the conditions of this experiment, the recommended dietary inclusion level of LRL ranges from 5% to 15%, and the 5% supplementation level of LRL yielded the optimal results.

## 6. Limitations

It should be noted that the slaughter analysis was conducted on a subset of five randomly selected lambs per group. While this sample size is consistent with common practices in the field and yielded statistically significant results, future studies with larger cohorts could provide even greater statistical power to confirm these findings.

## Figures and Tables

**Table 1:** Conventional nutrient content of LRL.

Items	Nutrient Content, %
DM	95.21
CP	13.31
EE	3.14
NDF	26.90
ADF	11.91
Ca	0.95
P	0.15

Notes: Nutritional levels are all measured values. Except for DM (measured after air drying), the rest are measured on a dry matter (DM) basis. DM, dry matter; CP, crude protein; EE, ether extract; NDF, neutral detergent fiber; ADF, acid detergent fiber.

**Table 2:** Percent composition and nutrient levels of experimental diets (DM basis).

Items	Control Group	LRL Groups		
		5%	10%	15%
Ingredients, %				
Corn	33.80	33.50	32.00	32.00
Wheat bran	9.00	6.20	6.00	5.90
Cottonseed meal	13.70	11.00	8.80	6.60
LRL	0.00	5.00	10.00	15.00
Corn stalks	18.00	19.00	20.00	20.00
Alfalfa hay	20.50	20.30	18.20	15.50
Premix ^1^	5.00	5.00	5.00	5.00
Total	100.00	100.00	100.00	100.00
Nutritional level ^2^, %				
ME (MJ/kg)	11.06	11.07	11.00	11.06
DM	92.19	92.12	92.47	92.32
CP	13.36	13.13	13.46	13.26
EE	2.12	2.10	2.71	2.96
Ash	11.12	11.70	11.19	11.10
NDF	55.55	53.15	52.63	53.77
ADF	25.15	23.62	21.13	21.25
Ca	0.70	1.15	0.61	1.01
P	0.49	0.42	0.45	0.42

^1^ Each kg of premix contained: vitamin A, 150,000 IU; vitamin D_3,_ 56,500 IU; vitamin E, 8000 IU; Se (as sodium selenite), 14 mg; I (as potassium iodide), 58 mg; Cu (as copper sulfate), 290 mg; Mn (as manganese sulfate), 1925 mg; Zn (as zinc oxide), 2050 mg; Co (as cobalt sulfate), 24 mg. ^2^ ME is a calculated value; all other nutrient levels were measured values. ME, metabolizable energy; DM, dry matter; CP, crude protein; EE, ether extract; NDF, neutral detergent fiber; ADF, acid detergent fiber; Ca, calcium; P, phosphorus.

**Table 3:** Effect of LRL on rumen fermentation parameters of lambs (*n* = 5).

Items	Control Group	LRL Groups			SEM	*p* Value		
		5%	10%	15%		Treatment	Linear	Quadratic
pH	5.72	5.70	5.72	5.84	0.067	0.859	0.495	0.620
NH_3_-N (mg/dL)	26.13	27.65	27.59	28.11	0.600	0.680	0.283	0.700
Acetate (mmol/L)	88.50 ^b^	115.27 ^a^	103.34 ^ab^	101.11 ^ab^	3.944	0.027	0.356	0.061
Propionate (mmol/L)	27.34	33.35	33.06	28.33	1.525	0.401	0.833	0.101
Isobutyrate (mmol/L)	0.87	1.00	0.91	0.80	0.033	0.222	0.301	0.089
Butyrate (mmol/L)	20.43 ^b^	33.56 ^a^	28.11 ^ab^	24.53 ^b^	1.688	0.026	0.496	0.008
Isovalerate (mmol/L)	1.34 ^Bb^	2.62 ^Aa^	1.74 ^Bb^	1.45 ^Bb^	0.146	0.001	0.635	0.001
Valerate (mmol/L)	1.61	2.06	1.92	1.87	0.133	0.714	0.581	0.387
TVFA (mmol/L)	140.09 ^b^	186.40 ^a^	169.29 ^ab^	159.08 ^ab^	6.172	0.042	0.332	0.016
Acetate/Propionate	3.36	3.63	3.20	3.60	0.168	0.818	0.803	0.858

In the same row, values with no letter or the same letter superscripts indicate no significant difference (*p* > 0.05), while values with different lowercase letter superscripts are significantly different (*p* < 0.05), and values with different uppercase letter superscripts are highly significantly different (*p* < 0.01). The following tables are organized in the same way. SEM, standard error of the mean; NH_3_-N, ammonia nitrogen; TVFA, total volatile fatty acids.

**Table 4:** Effect of dietary LRL addition on rumen bacterial alpha diversity in lambs.

Items	Control Group	LRL Groups			SEM	*p* Value		
		5%	10%	15%		Treatment	Linear	Quadratic
ACE Index	1154.20 ^ab^	947.14 ^b^	912.38 ^b^	1283.64 ^a^	51.410	0.015	0.974	0.004
Chao1 Index	1158.67 ^ab^	951.74 ^b^	919.19 ^b^	1292.25 ^a^	51.814	0.016	0.998	0.004
Simpson Index	0.99	0.99	0.98	0.99	0.002	0.120	0.756	0.154
Shannon Index	8.09 ^ab^	7.70 ^bc^	7.26 ^c^	8.51 ^a^	0.157	0.018	0.991	0.011
Good Coverage	99.96	99.95	99.95	99.95	<0.001	0.094	0.212	0.540

In the same row, values with no letter or the same letter superscripts indicate no significant difference (*p* > 0.05), while values with different lowercase letter superscripts are significantly different (*p* < 0.05).

**Table 5:** Effect of dietary LRL supplementation on the relative abundance of rumen bacteria at the phylum level (% of total sequences).

Items	Control Group	LRL Groups			SEM	*p* Value		
		5%	10%	15%		Treatment	Linear	Quadratic
Firmicutes	54.51 ^ab^	76.20 ^b^	45.5 ^a^	53.86 ^ab^	3.590	0.012	0.821	0.030
Bacteroidota	38.13 ^a^	16.82 ^b^	48.91 ^a^	34.41 ^a^	3.634	0.012	0.904	0.098
Proteobacteria	1.71	2.12	1.52	2.88	0.385	0.629	0.458	0.534
Desulfobacterota	1.43 ^ab^	0.34 ^b^	0.38 ^b^	2.39 ^a^	0.293	0.020	0.683	0.004
Patescibacteria	1.04	1.69	1.41	0.82	0.167	0.281	0.969	0.059
Actinobacteriota	1.09	1.26	1.00	0.94	0.155	0.915	0.782	0.571
unclassified_Bacteria	0.27	0.35	0.04	2.24	0.447	0.258	0.253	0.168
Verrucomicrobiota	0.50	0.31	0.34	0.72	0.079	0.222	0.657	0.057
Fibrobacterota	0.26	0.04	0.31	0.47	0.075	0.268	0.432	0.085
Acidobacteriota	0.21	0.22	0.16	0.23	0.042	0.952	0.933	0.833

In the same row, values with no letter or the same letter superscripts indicate no sig-nificant difference (*p* > 0.05), while values with different lowercase letter superscripts are significantly different (*p* < 0.05).

**Table 6:** Effect of dietary LRL supplementation on the relative abundance of rumen bacteria at the genus level (% of total sequences).

Items	Control Group	LRL Groups			SEM	*p* Value		
		5%	10%	15%		Treatment	Linear	Quadratic
*Prevotella*	12.53 ^ab^	7.29 ^b^	21.08 ^a^	14.20 ^ab^	2.035	0.030	0.479	0.476
*Christensenellaceae_R_7_*group	10.89 ^ABab^	16.62 ^Aa^	6.39 ^Bb^	9.46 ^ABb^	1.265	0.033	0.467	0.086
*NK4A214_*group	5.70 ^Bc^	14.29 ^Aa^	12.07 ^Bb^	7.08 ^ABb^	1.191	0.017	0.142	0.004
*Ruminococcus*	4.86 ^b^	14.30 ^a^	9.27 ^ab^	3.29 ^b^	1.576	0.047	0.758	0.007
*Prevotellaceae_UCG_*001	7.17	1.38	12.30	1.58	2.418	0.378	0.663	0.876
*Uncultured_rumen_bacterium*	6.41	2.66	3.85	6.13	0.609	0.073	0.350	0.015
*Rikenellaceae_RC9_gut_*group	5.85 ^a^	1.65 ^b^	5.47 ^ab^	3.67 ^a^	0.601	0.044	0.145	0.145
*Lachnospiraceae_NK3A20_*group	2.77 ^a^	5.25 ^b^	1.81 ^a^	2.03 ^ab^	0.449	0.018	0.496	0.015
*Unclassified Prevotellaceae*	1.63	0.88	2.45	3.58	0.565	0.389	0.295	0.183
*Ruminococcus_gauvreauii_*group	0.98	4.48	0.17	1.59	0.604	0.071	0.742	0.105

In the same row, values with no letter or the same letter superscripts indicate no sig-nificant difference (*p* > 0.05), while values with different lowercase letter superscripts are significantly different (*p* < 0.05), and values with different uppercase letter superscripts are highly significantly different (*p* < 0.01).

**Table 7:** Effect of dietary LRL supplementation on slaughter performance and meat quality of lambs (*n* = 5).

Items		Control Group	LRL Groups			SEM	*p* Value		
			5%	10%	15%		Treatment	Linear	Quadratic
Live Weight Before Slaughter (kg)		44.86	44.82	43.96	46.04	0.403	0.358	0.681	0.254
Carcass Weight (kg)		21.44	22.20	21.64	22.32	0.285	0.677	0.384	0.972
Slaughter Rate (%)		47.82	49.53	49.30	48.40	0.318	0.635	0.460	0.298
Eye Muscle Area (cm^2^)		14.25	15.10	14.98	14.86	0.503	0.956	0.629	0.829
Water Loss Rate (%)		24.55	22.11	22.28	22.82	0.498	0.303	0.117	0.288
Meat Color	L*	35.28 ^Bc^	40.58 ^ABb^	44.46 ^Aab^	47.49 ^Aa^	1.290	0.001	<0.001	0.202
	a*	17.16 ^Aa^	11.15 ^Bb^	11.29 ^Bb^	10.93 ^Bb^	0.657	<0.001	<0.001	0.025
	b*	10.00	11.80	10.76	13.58	0.601	0.171	0.084	0.468
pH_45 min_		6.89	6.73	6.85	6.77	0.053	0.733	0.476	0.706
Moisture (%)		74.14	71.50	73.38	73.74	0.544	0.345	0.616	0.121

In the same row, values with no letter or the same letter superscripts indicate no sig-nificant difference (*p* > 0.05), while values with different lowercase letter superscripts are significantly different (*p* < 0.05), and values with different uppercase letter superscripts are highly significantly different (*p* < 0.01).

**Table 8:** Effect of dietary LRL supplementation on amino acid concentration in lamb (*n* = 5).

Items	Control Group	LRL Groups			SEM	*p* Value		
		5%	10%	15%		Treatment	Linear	Quadratic
Essential amino acids, EAAs								
Ile	37.35	48.85	46.25	50.17	2.884	0.417	0.122	0.766
Leu	87.33	111.71	122.32	107.70	6.034	0.225	0.087	0.352
Thr	36.64 ^Bb^	54.17 ^ABa^	52.54 ^ABa^	57.68 ^Aa^	2.615	0.009	0.001	0.598
Lys	68.18 ^b^	91.44 ^ab^	86.49 ^ab^	109.60 ^a^	6.604	0.043	0.046	0.626
Trp	11.03	13.63	15.14	13.90	0.839	0.388	0.126	0.635
Phe	47.71	61.41	57.07	55.53	3.618	0.634	0.352	0.396
Val	45.93	68.58	61.03	71.21	4.247	0.141	0.037	0.711
Met	34.53	41.56	39.88	39.30	2.208	0.739	0.377	0.529
TEAA	368.70	515.53	488.63	468.32	24.628	0.163	0.067	0.184
Non-essential amino acids, NEAAs								
Asp	28.07 ^Bb^	20.61 ^Bb^	23.39 ^Bb^	49.34 ^Aa^	3.451	0.004	0.096	0.001
Ser	69.62 ^b^	84.98 ^ab^	84.81 ^ab^	98.24 ^a^	4.435	0.37	0.033	0.611
Glu	83.31 ^Aa^	94.06 ^Aa^	87.17 ^Aa^	40.80 ^Bb^	6.380	0.003	0.051	0.002
Pro	29.87 ^Bb^	43.93 ^Aa^	44.05 ^Aa^	49.22 ^Aa^	2.230	0.005	<0.001	0.894
Gly	58.07 ^Bb^	69.11 ^ABb^	85.79 ^Aa^	83.77 ^Aa^	3.240	0.001	<0.001	0.474
Ala	253.20 ^Bc^	301.69 ^ABab^	279.18 ^ABbc^	337.43 ^Aa^	10.020	0.010	0.004	0.384
Tyr	45.11	43.08	54.09	50.73	3.735	0.743	0.522	0.683
His	110.07 ^ab^	122.66 ^ab^	142.76 ^a^	103.99 ^b^	6.056	0.033	0.551	0.102
Arg	63.46 ^a^	83.70 ^b^	86.02 ^b^	86.90 ^b^	3.505	0.039	0.006	0.527
TNEAA	1074.34 ^b^	1231.30 ^ab^	1285.23 ^a^	1225.36 ^ab^	32.922	0.119	0.032	0.366
DAA	111.38 ^ab^	118.79 ^a^	115.65 ^a^	89.39 ^b^	4.289	0.049	0.225	0.016
SAA	458.42 ^b^	576.45 ^a^	564.86 ^a^	612.40 ^a^	20.088	0.026	0.004	0.842
TAA	1443.04	1746.62	1773.46	1693.08	55.778	0.131	0.039	0.260

In the same row, values with no letter or the same letter superscripts indicate no sig-nificant difference (*p* > 0.05), while values with different lowercase letter superscripts are significantly different (*p* < 0.05), and values with different uppercase letter superscripts are highly significantly different (*p* < 0.01). TEAA, total essential amino acids; TNEAA, total non-essential amino acid; DAA, delicious amino acids; SAA, sweet amino acids; TAA, total amino acids.

**Table 9:** Effect of LRL on percent fatty acid content in lamb (*n* = 5).

Items	Control Group	LRL Groups			SEM	*p* Value		
		5%	10%	15%		Treatment	Linear	Quadratic
C10:0	0.28	0.22	0.18	0.28	0.026	0.544	0.613	0.304
C12:0	0.21	0.38	0.30	0.51	0.062	0.368	0.146	0.677
C14:0	3.32	3.98	3.49	4.16	0.282	0.727	0.416	0.959
C14:1	0.08	0.33	0.42	0.21	0.058	0.165	0.130	0.148
C15:0	0.48	0.48	0.48	0.53	0.041	0.965	0.773	0.697
C16:0	26.80	26.58	26.64	26.29	0.211	0.877	0.501	0.772
C16:1	1.38	2.61	2.83	2.06	0.269	0.231	0.140	0.180
C17:0	1.32	1.23	1.29	1.44	0.062	0.701	0.731	0.271
C18:0	23.01 ^a^	19.13 ^b^	20.07 ^ab^	20.77 ^ab^	0.614	0.039	0.079	0.114
C18:1n9c	37.65	37.80	36.41	35.84	0.646	0.687	0.377	0.466
C18:2n6c	4.08	2.99	3.20	3.42	0.235	0.421	0.203	0.289
C18:3n3	0.40	0.51	0.34	0.52	0.076	0.839	0.773	0.948
C20:0	0.12	0.14	0.13	0.15	0.011	0.855	0.425	0.968
C20:1	0.07 ^b^	0.11 ^ab^	0.12 ^a^	0.08 ^ab^	0.009	0.031	0.109	0.096
C20:4n6	0.21	0.21	0.19	0.17	0.021	0.918	0.596	0.661
SFAs	55.54	52.14	52.59	54.13	0.632	0.215	0.179	0.104
MUFAs	39.17	40.85	39.78	38.19	0.893	0.791	0.907	0.325
PUFAs	4.68	3.70	3.73	4.10	0.248	0.497	0.249	0.325

In the same row, values with no letter or the same letter superscripts indicate no sig-nificant difference (*p* > 0.05), while values with different lowercase letter superscripts are significantly different (*p* < 0.05). SFAs, saturated fatty acids; MUFAs, monounsaturated fatty acids; PUFAs, polyunsaturated fatty acids.

## Data Availability

All data was uploaded to Figshare: https://figshare.com/s/50e18df156f8b41aa7fe (accessed on: 13 September 2025), https://doi.org/10.6084/m9.figshare.30119413.

## Supplementary Materials

The following supporting information can be downloaded at: https://www.mdpi.com/article/10.3390/ani15213118/s1, Figure S1: Rarefaction curves of 16S rRNA gene sequencing for all samples.
