# Supplementary figures and images for: Effects of Different Levels of Lycium ruthenicum Leaves on Rumen Fermentation, Amino Acids, Fatty Acids and Rumen Bacterial Diversity in Sheep
Source: Animals (Basel). 2025 Oct 27;15(21):3118. doi: 10.3390/ani15213118 (PMC12608922; doi:10.3390/ani15213118)

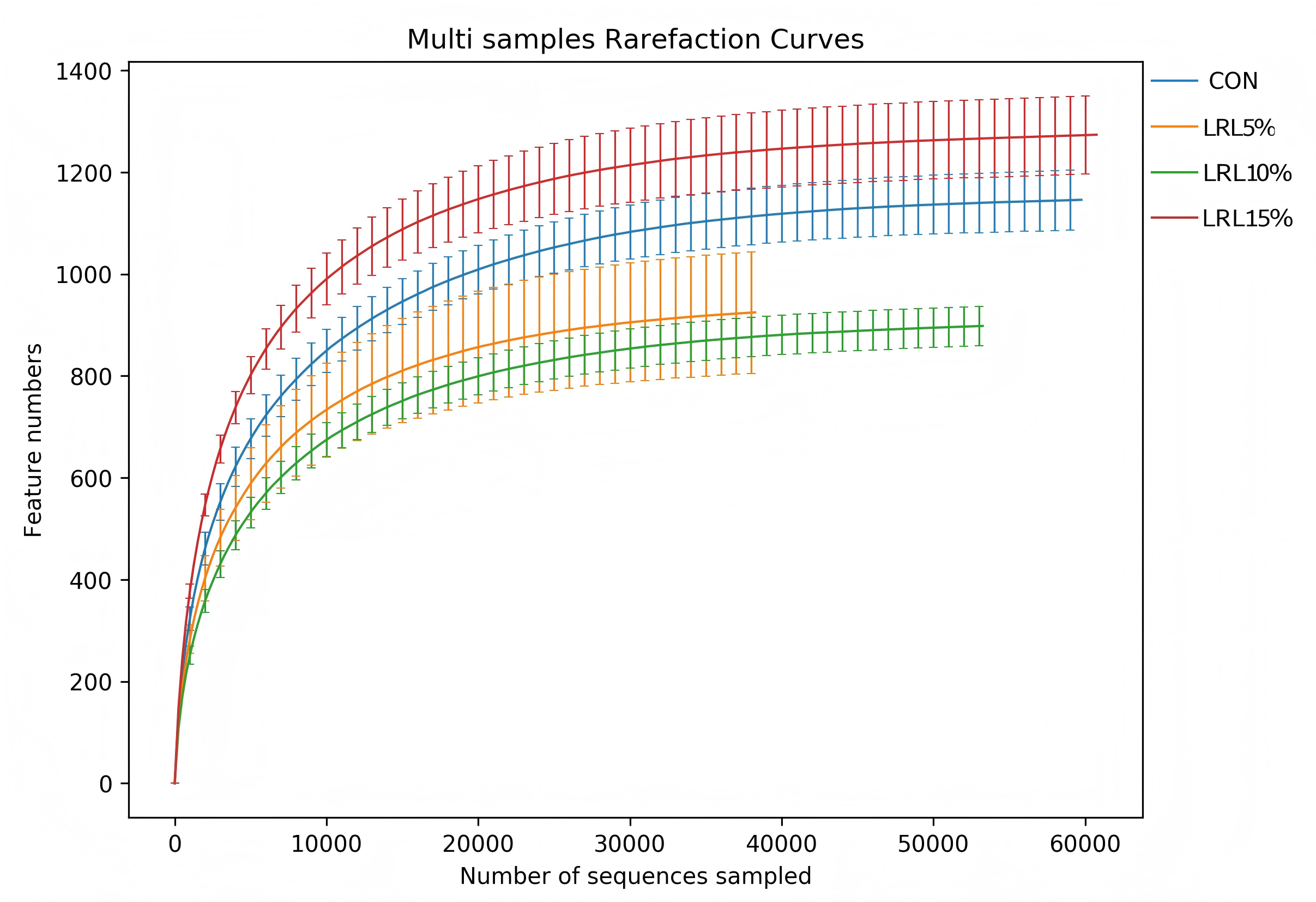

Supplement: Supplementary file 1 [file animals-15-03118-s001.zip › animals-3900646-supplementary.png]
